# American Medical Association

**Published:** 1879-04-20

**Authors:** 


### EDITORIAL AND MISCELLANEOUS.

### *AMERICAN MEDICAL ASSOCIATION.*

Arrangements for the meeting of the American Medical Association, in the city of Atlanta on the 6th of May, have taken very decided shape since our last issue.

We learn that reduced rates of transportation will be allowed upon the various lines of railroads terminating here, and that every reasonable facility will be extended to those desiring to attend from the entire country, North, South, East and West.

In addition, we are assured that ample and convenient halls will be provided for the general meetings of the Body, and for the sessions of the various Sections.

As we go to press the programme in detail has not been definitely announced. It is most probable that the regular meetings of the Body will be held in the Representative Hall of the Capitol.

We learn from Dr. Logan, Chairman of the Committee of Arrangements, that he has received the usual notice of a number of valuable papers to be presented to the various Sections, and can promise a highly interesting "Programme."

We are also confident that the social features of the occasion will do no discredit to the profession or to the city, so that we feel no hesitancy in extending a hearty welcome to all who can attend.

*The Association of Medical Colleges*, appointed in Atlanta for May the 2d, will hold its meetings, we learn, in the Senate Chamber.

*The National Board of Health* will also convene in Atlanta on May the first.

